# Tracheal tubes lubricated with water to reduce sore throat after intubation: A randomized non-inferiority trial

**DOI:** 10.1371/journal.pone.0204846

**Published:** 2018-10-04

**Authors:** Eugene Kim, Seong Mi Yang, Sang Gyu Kwak, Seoyeong Park, Jae-Hyon Bahk, Jeong-Hwa Seo

**Affiliations:** 1 Department of Anesthesiology and Pain Medicine, Seoul National University Hospital, Seoul National University College of Medicine, Seoul, Republic of Korea; 2 Department of Medical Statistics, Daegu Catholic University College of Medicine, Daegu, Republic of Korea; Public Library of Science, UNITED KINGDOM

## Abstract

**Background:**

Sore throat is common after tracheal intubation. Water can be used to lubricate tracheal tubes, but its benefit has not been validated. We thus did a randomised non-inferiority trial to test the hypothesis that a tube lubricated with water does not reduce sore throat after tracheal intubation.

**Methods:**

We randomized female or male patients (n = 296) undergoing surgery in the ears or eyes to receive either a tube lubricated with water or a tube without lubrication for intubation. We assessed sore throat at 0, 2, 4, and 24 h after surgery; pharyngeal injury at 2 and 24 h after surgery; and respiratory infections within 7 days after surgery. For the incidence of sore throat within 24 h after surgery (primary outcome), the two-sided 90% confidence interval of the risk difference was compared with the prespecified non-inferiority margin of 15%. Other outcomes were analyzed with two-sided superiority tests.

**Results:**

The incidence of sore throat within 24 h after surgery was 80/147 (54.4%) in the non-lubricated tube group and 83/149 (55.7%) in the water-lubricated tube group (risk difference -1.3%, 90% confidence interval -10.9% to 8.3%). Because the confidence interval was below the non-inferiority margin, the incidence of sore throat was not higher in the non-lubricated tube group than in the water-lubricated tube group. There was no significant association between groups in the sore throat, pharyngeal injury, and respiratory infection at each assessment time.

**Conclusions:**

The tube lubricated with water did not reduce sore throat and pharyngeal injury after tracheal intubation compared to the tube without lubrication.

## Introduction

Sore throat is common after tracheal intubation [1–21]. Various drugs [7,9,12,13,15,16,19] or lubricants [5,10,14,17,18] have been introduced to reduce the sore throat although some methods seem to be applied without evidence. Moreover, these pharmacological interventions may not only affect the sore throat but also cause side effects.

Water has lubricating properties without pharmacological side effects and thus can be safely used to lubricate medical devices so that it may decrease trauma during manipulation of the devices [9,10,17,18,22–25]. Water is also conventionally used for lubricating tracheal tubes in order to reduce sore throat or airway injury associated with intubation, but its benefit has not been validated in tracheal tubes.

To lubricate a tracheal tube with water before intubation, the distal portion of the tube can be put into a water bottle, or some water can be applied to the external surface of the tube. However, such treatment may contaminate the tube and thus lead to respiratory infection after tracheal intubation. We thus performed a randomized non-inferiority trial to test the hypothesis that a tube lubricated with water does not reduce sore throat after tracheal intubation compared to a tube without lubrication in surgical patients undergoing general anesthesia. We also compared postoperative airway injury and respiratory infection between the use of a water-lubricated tube and the use of a non-lubricated tube for tracheal intubation.

## Materials and methods

### Patients

This trial was approved by the Institutional Review Board of Seoul National University Hospital (H-1506-125-684) and was compliant to the CONSORT checklist (S1 Checklist). Its protocol was registered at ClinicalTrials.gov (NCT02492646) and published [26]. Written informed consent was obtained from each patient prior to the study. We enrolled female or male patients aged 20–80 years with American Society of Anesthesiologists physical status of I–III, and undergoing elective surgery in the ears or eyes under general anaesthesia with orotracheal intubation between August 2015 and January 2017. We excluded patients with preoperative sore throat, hoarseness, respiratory infections, gastro-esophageal reflux, history of airway surgery, abnormality in the upper airway, and anticipated difficult intubation such as Mallampati class of ≥ III or thyromental distance of < 6.5 cm.

Patients were randomized to receive either a tube lubricated with water or a tube without lubrication for tracheal intubation. An unrelated assistant created a randomization in a 1:1 ratio with random block sizes of 4 or 6 using an online tool (http://www.randomization.com) and concealed the allocation sequence in sealed opaque envelopes.

### Preparation of tracheal tubes

A nurse unaware of the study protocol prepared a disposable tracheal tube made of polyvinyl chloride (Unomedical, Kedah, Malaysia) in an aseptic manner. The inner diameter of the tube was 7.0 mm for women and 7.5 mm for men. In the water-lubricated tube group, the tube was placed in a 1-L bottle of sterile saline (Daihan Pharm, Seoul, Korea) with a room temperature of 22 ± 1°C according to the conventional protocol at our institution. The distal half of the tube was immersed in the water. In the non-lubricated tube group, the tube was kept in the sterile package without any treatment until intubation. The tube cuff was completely deflated before intubation.

### Anesthesia

Without premedication, patients were monitored with pulse oximetry, electrocardiography, non-invasive blood pressure, bispectral index (A-2000 XP; Aspect Medical Systems, Newton, MA, USA) and acceleromyography (TOF-watch; Organon Ltd., Dublin, Ireland). Patients received forced-air warming with a full-body blanket (Model 300; 3M Bair Hugger, Eden Prairie, MN, USA) at a set temperature of 43°C. In the supine position, the patient’s head was placed on an incompressible pillow of 7-cm height.

Anesthesia was induced by intravenous propofol 1.5–2.0 mg kg^-1^ and fentanyl 1 μg kg^-1^. The acceleromyograph was calibrated and stabilized by a 50-Hz tetanic stimulation for 5 sec followed by serial train-of-four (TOF) measurements within a 5% variation. [27] Rocuronium 0.6–0.8 mg kg^-1^ was given and TOF counts were monitored every 15 s at the adductor pollicis muscle.

At a TOF count of 0 and bispectral index of < 60, an investigator (EK) intubated the patient’s trachea using either a water-lubricated or a non-lubricated tube via direct laryngoscopy with a Macintosh 3 or 4 blade. If the intubation failed, it was retried after the tube was shaped like a hockey-stick with a stylet. Tracheal intubation was achieved with video laryngoscopy (UESCOPE; UM Medical Devices, Newton, MA, USA) or fiberoptic bronchoscopy (LF-GP; Olympus Optical Co., Tokyo, Japan) after two failures of direct laryngoscopy. After intubation, the intracuff pressure of the tube was adjusted to less than 25 cmH_2_O (VBM Medizintechnik GmBH, Sulz am Neckar, Germany) [6].

Anesthetists unrelated to the study maintained anesthesia with 1.0–1.5 minimum alveolar concentration of desflurane to obtain a bispectral index of < 60. Rocuronium 0.2–0.4 mg kg^-1^ was given at a TOF count of ≥ 1. The patient’s lungs were ventilated with a tidal volume of 6–8 mg∙kg^-1^, positive end-expiratory pressure of 5–10 cmH_2_O, respiratory rate of 10–16 min^-1^, and inspired oxygen fraction of 0.4–0.6 (Avance; GE Datex-Ohmeda, Munich, Germany). A mixture of oxygen and air was supplied with a 2 L∙min^-1^ of gas flow.

After surgery, pyridostigmine 0.3 mg kg^-1^ and glycopyrrolate 0.01 mg kg^-1^ were administered at a TOF count of ≥ 2. An investigator (J-HS) extubated the trachea when the patient had spontaneous breathing and responses to verbal commands at a TOF ratio of > 0.9 and bispectral index of > 60. Patients were transferred to the post-anesthesia room. Postoperative pain was assessed with a 10-point scale (0, no pain; 10, worst pain imaginable) and fentanyl 25–50 μg was given at the pain score of > 4 or at the patient’s request. Patients were discharged from the hospital when they were able to walk and eat food without severe pain or complications.

### Outcomes

Before surgery, an investigator (SMY) evaluated the patient’s airway with the thyromental distance and Mallampati class. Before intubation, another investigator (EK) performed manual ventilation in the sniffing position without an oropharyngeal airway and graded it as easy, moderate, or difficult ventilation. During intubation, the investigator assessed the Cormack-Lehane grade, the time and number of attempts for intubation, and the resistance of the tube as it passed the glottis. The intubation time was defined as the interval between the insertion of the laryngoscopic blade into the mouth and the inflation of the tube cuff after successful intubation [19]. The resistance of the tube was graded as none, mild, moderate, or severe resistance [20,21]. Mean blood pressure and heart rate were measured immediately before and one minute after intubation. After extubation, an investigator (J-HS) checked for blood in the oral cavity or on the tube surface and recorded the interval between the discontinuation of desflurane and tracheal extubation.

At 0, 2, 4, and 24 h after surgery, an investigator (SMY) asked patients two questions: “Do you have any discomfort after surgery?” and then “Do you have a sore throat?”. Sore throat was graded into four severity levels as follows: no symptom, mild throat pain only upon the second question, moderate pain in response to the first question, and severe pain with change of voice or hoarseness [12,15,17–19]. At 2 and 24 h after surgery, the investigator examined the pharynx using a penlight and tongue depressor and recorded the site and type of injury. The site was classified as uvula, posterior wall, tonsillar pillar or fossa, and vallecula; and the type as hyperemia, edema, hematoma, and others [20,21]. Seven days after surgery, the investigator asked patients about symptoms or medications of postoperative respiratory infections. The symptom was categorized as cough, sputum, rhinorrhea, sore tongue, myalgia, fever, and others. If the patient was discharged from the hospital, they were contacted by phone.

Patients and investigators were blinded to group assignment except for the intubator (EK). The primary outcome was the incidence of sore throat within 24 h after surgery. Secondary outcomes were the resistance while the tracheal tube passed the glottis, sore throat, pharyngeal injury, and respiratory infection at each assessment time.

### Statistical analysis

In a previous study [19], 57% of patients complained of sore throat within 24 h after tracheal intubation with water-lubricated tubes. Considering a non-inferiority margin of 15%, 135 patients were needed in each group with a power of 0.8 and one-sided α of 0.05 (PASS version 11.0; NCSS, Kaysville, UT, USA).

Continuous variables were presented as mean ± SD or median (IQR) after checking the normality with the Kolmogorov-Smirnov test and analyzed with two or paired sample t-tests or with Mann-Whitney U or Wilcoxon signed-rank tests. Categorical variables were the number of patients and were compared with chi-squared test or with Fisher’s exact test if two of the frequency cell were smaller than 5. For the incidence of postoperative sore throat (primary outcome), the two-sided 90% (1–2α) confidence interval of the risk difference was compared with the pre-specified non-inferiority margin of 15%. Non-inferiority was accepted for the non-lubricated tube over the water-lubricated tube if the upper limit of the confidence interval was less than the non-inferiority margin. Secondary outcomes were analyzed with two-sided superiority tests. A P-value of < 0.05 was considered significant. A medical statistician (SGK) unrelated to data collection analyzed the outcomes with SPSS (version 21.0; IBM, Chicago, IL, USA) and STATA (Special Edition 14.2; Stata Corporation, College Station, TX, USA).

## Results

**Fig 1:**
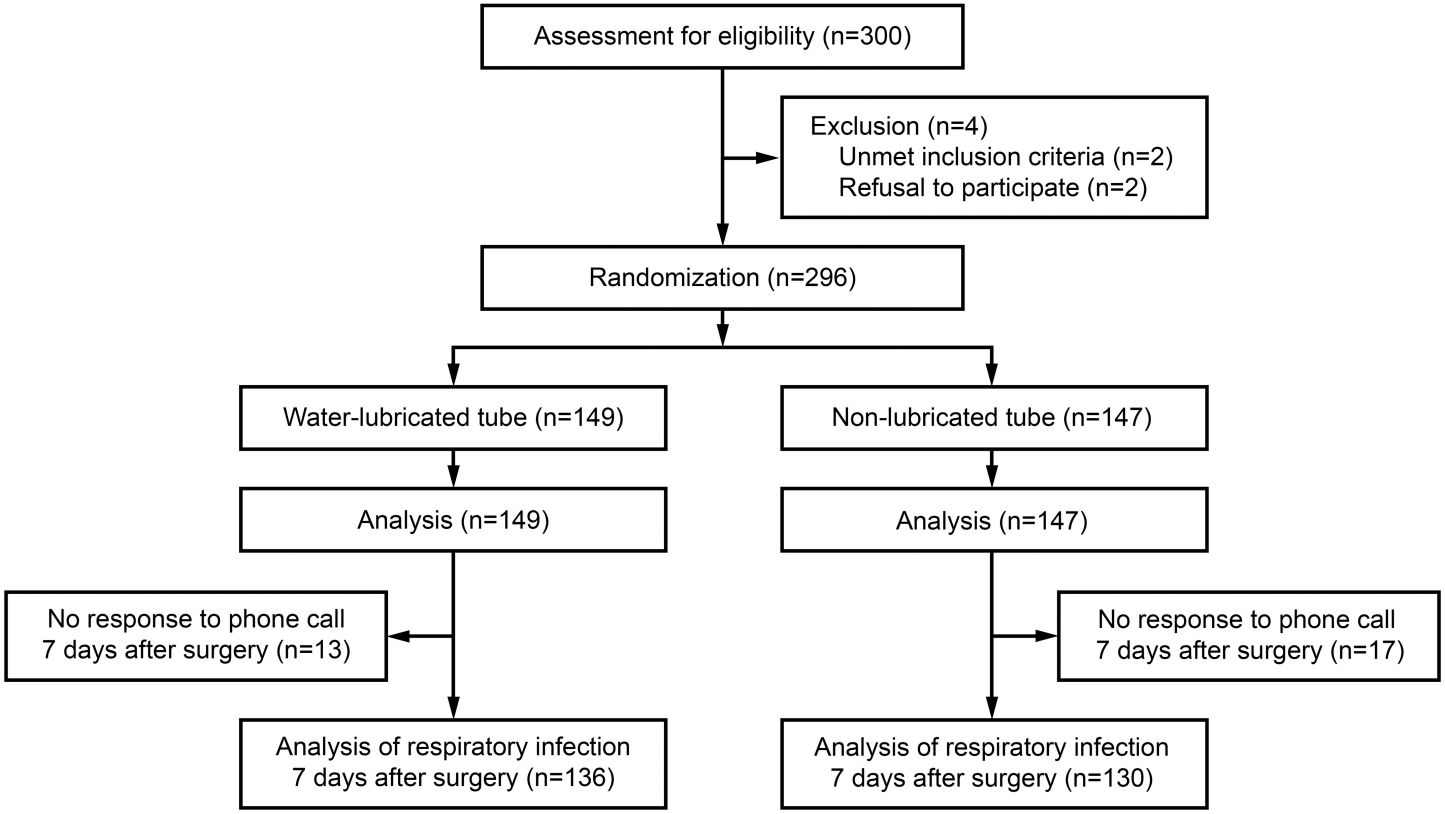
CONSORT flow diagram. At seven days after surgery, patients were asked by phone call whether they had respiratory infection since the surgery.

After screening 300 patients, 296 patients were randomized to the water-lubricated (n = 149) or non-lubricated (n = 147) tube groups (Fig 1). There was no significant association in patient characteristics (Table 1) and preoperative airway evaluation (Table 2).

**Table 1:** Characteristics of patients, anesthesia, and surgery.

	Water-lubricated tube (n = 149)	Non-lubricated tube (n = 147)
Age (year)	53.4 ± 13.8	54.5 ± 14.5
Sex		
Female	71 (47.7%)	79 (53.7%)
Male	78 (52.3%)	68 (46.3)
Weight (kg)	64.9 ± 11.9	65.5 ± 12.3
Height (cm)	163.3 ± 8.8	162.0 ± 8.0
Body mass index (kg m^-2^)	24.2 ± 3.3	24.9 ± 4.4
ASA physical status		
I	66 (44.3%)	59 (40.1%)
II	83 (55.7%)	86 (58.5%)
III	0	2 (1.4%)
Medical conditions		
Hypertension	39 (26.2%)	41 (27.9%)
Diabetes	25 (16.8%)	23 (15.6%)
Asthma	7 (4.7%)	4 (2.7%)
Angina	5 (3.4%)	7 (4.8%)
Hepatitis	2 (1.3%)	2 (1.4%)
Normal		
Type of surgery		
Ear	84 (56.4%)	77 (52.4%)
Eye	65 (43.6%)	70 (47.6%)
Amount of anesthetic drugs		
Propofol (mg)	117.5 ± 21.5	118.5 ± 22.2
Fentanyl (μg)	69.4 ± 12.7	70.1 ± 13.1
Rocuronium (mg)	81.8 ± 15.0	82.5 ± 15.5
Duration of intervention		
Intubation (min)	145.5 ± 82.8	143.1 ± 86.3
Surgery (min)	117.1 ± 73.2	115.6 ± 79.6
Anesthesia (min)	155.8 ± 81.3	152.3 ± 87.1
Hospitalization (day)	3.1 ± 1.6	3.5 ± 3.4

Values are mean ± SD or number of patients (percetage). ASA, American Society of Anesthesiologists.

**Table 2:** Outcomes before, during, and after tracheal intubation.

	Water-lubricated tube (n = 149)	Non-lubricated tube (n = 147)	P value
Thyromental distance (mm)	72.7 ± 6.1	72.3 ± 5.1	0.570
Mallampati class			0.354
I	61 (40.9%)	49 (33.3%)	
II	82 (55.0%)	93 (63.3%)	
III	6 (4.0%)	5 (3.4%)	
Mask ventilation			0.759
Easy	118 (79.2%)	112 (76.2%)	
Moderate	26 (17.4%)	28 (19.0%)	
Difficult	5 (3.4%)	7 (4.8%)	
Cormack-Lehane grade			>0.999
I	83 (55.7%)	81 (55.1%)	
II	62 (41.6%)	62 (42.2%)	
III	4 (2.7%)	4 (2.7%)	
Resistance during intubation			0.001
None	136 (91.3%)	112 (76.2%)	
Mild	13 (8.7%)	33 (22.4%)	
Moderate	0	2 (1.4%)	
Intubation time (sec)	15.3 ± 10.2	14.6 ± 10.3	0.562
Number of intubation attempts			0.244
1	147 (98.7%)	142 (96.6%)	
2	2 (1.3%)	5 (3.4%)	
Mean blood pressure before intubation (mmHg)	71.0 ± 14.2	70.0 ± 13.2	0.508
Mean blood pressure after intubation (mmHg)	99.0 ± 26.5	94.0 ± 21.2	0.076
Heart rate before intubation (beats min^-1^)	67.0 ± 12.9	67.1 ± 12.5	0.952
Heart rate after intubation (beats min^-1^)	89.5 ± 17.5	86.4 ± 15.1	0.100
Extubation time (min)	7.9 ± 4.6	7.4 ± 3.9	0.332
Blood in the oral cavity after extubation	18 (12.1%)	22 (15.0%)	0.468
Blood on the tube surface after extubation	21 (14.1%)	29 (19.7%)	0.196
Postoperative analgesic medication	33 (22.1%)	30 (20.4%)	0.715
Postoperative respiratory infection^a^	48 (35.3%)	36 (27.7%)	0.190
Cough	10 (7.4%)	11 (8.5%)	
Sputum	24 (17.6%)	19 (14.6%)	
Rhinorrhea	6 (4.4%)	7 (5.4%)	
Sore tongue	2 (1.5%)	4 (3.1%)	
Myalgia	3 (2.2%)	1 (0.8%)	
Fever	2 (1.5%)	0	
Medication	6 (4.4%)	4 (3.1%)	

Values are mean ± SD or number of patients (percentage). Continuous variables were compared with two-sample t-test. Cormack-Lehane grade and resistance during intubation were compared with Fisher’s exact test, and the other categorical variables were analyzed with chi-squared test.

^a^Checked in 136 patient in the water-lubricated tube group and 130 patients in the non-lubricated tube group.

We achieved tracheal intubation with direct laryngoscopy at the first or second attempts in all patients and thus did not use the video laryngoscope or fiberoptic bronchoscope. During intubation, the tube passed the glottis with lower resistance in the water-lubricated tube group than in the non-lubricated tube group (Table 2; P = 0.001 by Fisher’s exact test). No significant association was found in the time or number of attempts for intubation. Mean blood pressure and heart rate significantly increased after tracheal intubation in both groups (P < 0.001 in each group, paired sample t-test), but did not differ between groups before and after intubation (Table 2).

**Fig 2:**
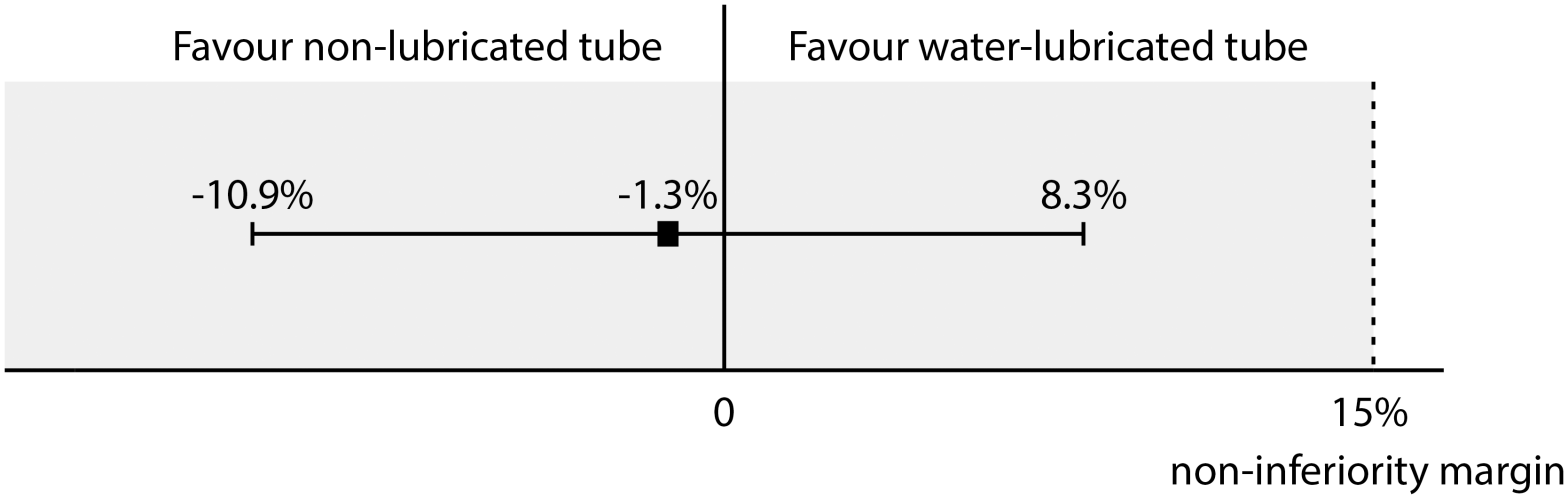
The risk difference (square) with 90% confidence interval (bar) for the incidence of sore throat within 24 h after surgery. The upper limit of the confidence interval is less than the pre-specified non-inferiority margin. This means that the incidence of sore throat is not higher in the non-lubricated tube group than in the water-lubricated tube group.

**Fig 3:**
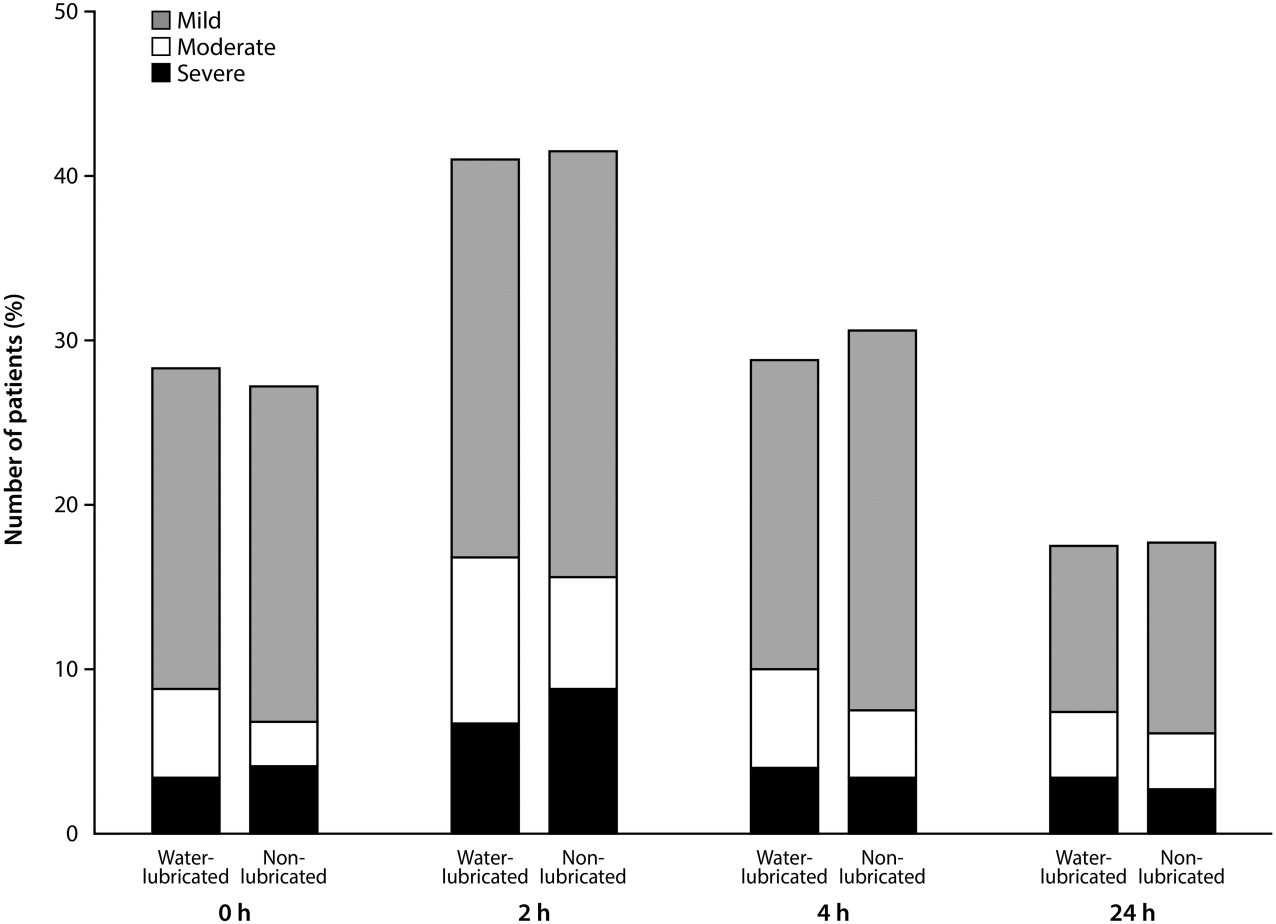
The incidence and severity of sore throat at 0, 2, 4, and 24 h after surgery in the water-lubricated and non-lubricated tube groups. P values are 0.851, 0.922, 0.741, and 0.957 at 0, 2, 4, and 24 h after surgery, respectively, by chi-squared tests.

**Fig 4:**
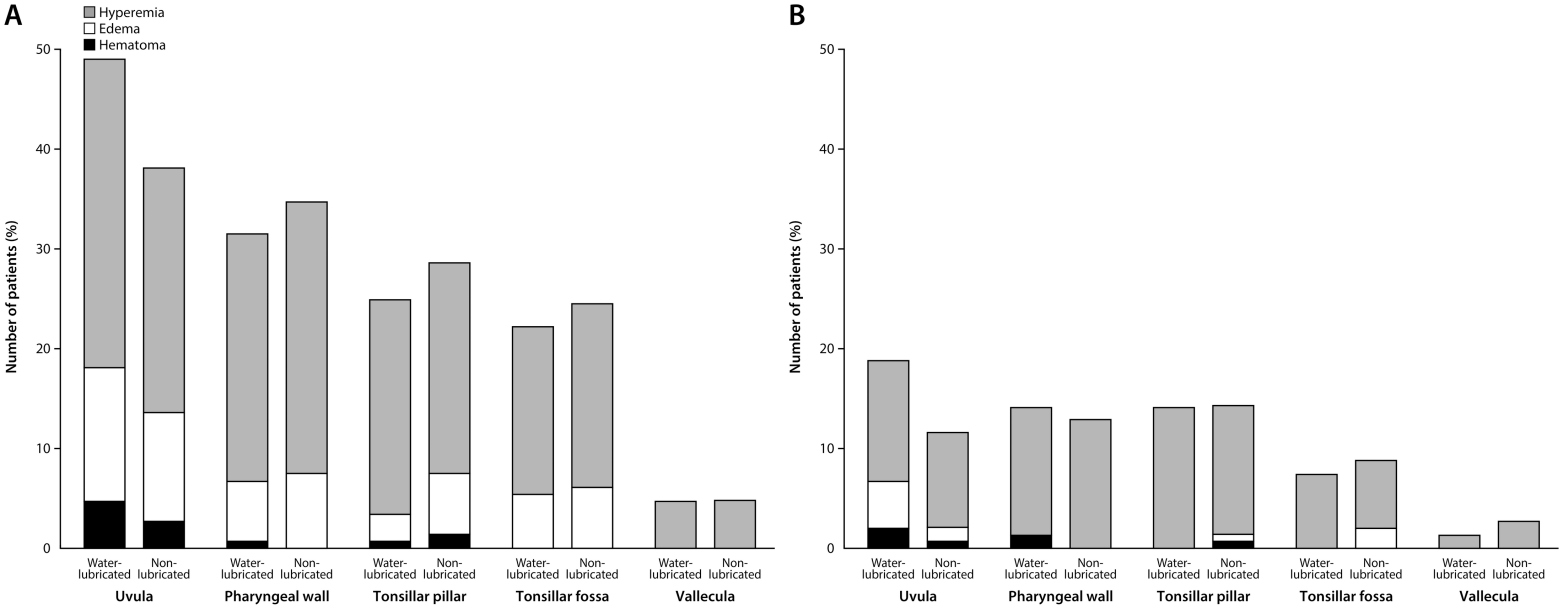
The site and type of pharyngeal injury at 2 (A) and 24 h (B) after surgery in the water-lubricated and non-lubricated tube groups. At 2 h after surgery (A), P values are 0.059, 0.565, 0.467, 0.634, and 0.979 in the uvula, pharyngeal wall, tonsillar pillar, tonsillar fossa, and vallecula, respectively, by chi-squared tests. At 24 h after surgery (B), P values are 0.083, 0.769, 0.962, 0.645, and 0.4000 in the same order as the fig A.

Within 24 h after surgery, sore throat was observed in 80 patients (54.4%) of the non-lubricated tube group and in 83 patients (55.7%) of the water-lubricated tube group (Fig 2; risk difference -1.3%, 90% confidence interval -10.9% to 8.3%). Because the upper limit of the 90% confidence interval was less than the non-inferiority margin of 15%, non-inferiority was accepted for the non-lubricated tube over the water-lubricated tube. Therefore, the incidence of sore throat was not higher in the non-lubricated tube group than in the water-lubricated tube group. There was no significant association between groups in the incidence or severity of sore throat at 0, 2, 4 and 24 h after surgery (Fig 3) and in the site and type of pharyngeal injury at 2 and 24 h after surgery (Fig 4).

Seven days after surgery, 13 patients (9.6%) in the water-lubricated tube group and 17 patients (13.1%) in the non-lubricated tube group did not respond to phone call (Fig 1). We thus asked 136 patients (91.3%) in the water-lubricated tube group and 130 patients (88.4%) in the non-lubricated tube group about symptoms or medications for respiratory infections (Fig 1) and observed no significant association between groups (Table 2). In the water-lubricated tube group, we found two patients with cough and sputum, two patients with cough with rhinorrhea, one patient with cough and medication, one with sputum and medication, and one with rhinorrhea and medications. In the non-lubricated tube group, we found one patient with cough, sputum, and rhinorrhea, one patient with cough and sputum, three patients with cough and rhinorrhea, one with cough and myalgia, one with cough and medication, one with sputum and rhinorrhea, and one with sputum and medication.

## Discussion

Water can reduce the friction between the tracheal tube and airway tissues during intubation because of its lubricating effect [22–25]. In our study, the water-lubricated tube passed the glottis with lower resistance than the non-lubricated tube although the resistance was assessed subjectively by the unblinded investigator. However, no significant association was found in the time or number of attempts for intubation and hemodynamic changes during intubation. Therefore, the water-lubricated tube may neither improve performance nor reduce noxious stimuli during tracheal intubation.

Sore throat is reported in up to 90% of patients within 24 h after general anesthesia [1,2,4–21]. Its main cause is known to be inflammation of the airway tissues damaged by intubation [5,6,12–14,16–19]. Therefore, the sore throat is known to be attenuated by steroids such as dexamethasone [13,16] and bethamethasone [5, 14] or non-steroidal drugs such as benzydamine hydrochloride [12, 17, 18], ketoprofen [7], aspirin [12], ketamine [15], and magnesium [19] that have topical [5,12,14,15,17–19] or systemic [7,13,16] anti-inflammatory effects. However, lubricants without anti-inflammatory actions may be ineffective [6,14] or even harmful [1,2] for the sore throat. Because water has only lubricating but not anti-inflammatory effects, it was unlikely to reduce postoperative sore throat in our study.

Tracheal tubes can damage upper airway tissues during intubation [6, 20, 21, 28–30]. The incidences of airway injuries were reported to be 33% in the larynx, 19% in the pharynx, and 15% in the trachea [28]. Although laryngeal injury is most common during intubation, [6,20,21,28] laryngoscopic examination may cause not only discomfort [31] but also sore throat [32,33] in awake patients. We thus only examined pharyngeal injury and found no significant association between groups. Because pharyngeal injury is associated with sore throat [28,34,35], it may also explain the similar incidence of postoperative sore throat between groups in our study.

Mechanical ventilation may cause respiratory infection [36–38]. The infection is associated with the duration of the ventilation [37], and the tracheal tube is a major infection source [36–40]. Patients in our study received sterile tracheal tubes and then mechanical ventilation for less than 4 h. As a result, we found no significant association in postoperative respiratory infections between groups, although this might be underpowered due to missing data. However, any external treatment could contaminate the tracheal tube and thus should be avoided unless it has definite advantages.

This study has limitations. Because sore throat is a subjective symptom, its assessment may differ according to the questioning method [2,3,6]. To minimize bias in our study, one blinded assessor interviewed all patients with the same questions and graded the severity based on previous studies [12,15,17–19]. Furthermore, we only evaluated symptoms but not pathogens or protein analysis for respiratory infections because most microorganisms cultured on the tube are known to be normal flora in the oropharynx [36,39,40,41]. In addition, we only studied tracheal tubes made of polyvinyl chloride, so our findings may not be extrapolated to other types of tubes.

In conclusion, the tube lubricated with water did not reduce sore throat and airway injury after tracheal intubation compared to the tube without lubrication. Therefore, it seems unnecessary to lubricate the tracheal tube with water before intubation.

## Supporting information

S1 Checklist CONSORT checklist. (DOCX)

S1 Protocol Study protocol (Korean). (DOCX)

S2 Protocol Study protocol (English). (DOCX)

S1 Dataset Dataset of the study. (XLSX)
