# Supplementary material for: Tracheal tubes lubricated with water to reduce sore throat after intubation: A randomized non-inferiority trial
Source: PLoS One. 2018 Oct 4;13(10):e0204846. doi: 10.1371/journal.pone.0204846 (PMC6171884; doi:10.1371/journal.pone.0204846)
Supplement: S1 Protocol — (DOCX) [file pone.0204846.s002.docx]

# **연구계획서**

| 기관 튜브의 관습적 식염수 수침 전처치가 기관 삽관 후 합병증에 미치는 영향  (영문 : Effect of normal saline immersing of endotracheal tube on post-intubation complications: A prospective randomized study) |
| --- |

**Version No : 2.0**

서울대학교 의과대학 마취과학 교실

부교수 서 정 화

**Protocol outline**

| 연구제목 | (국문) 기관 튜브의 관습적 식염수 수침 전처치가 기관 삽관 후 합병증에 미치는 영향 |
| --- | --- |
|  | (영문) Effect of normal saline immersing of endotracheal tube on post-intubation complications: A prospective randomized study |
| 책임연구자 | 마취통증의학과 서정화 부교수 |
| 연구비 지원기관 | 없음 |

| 연구 목적 | 기관내 삽관전에 관습적으로 행해지는 기관튜브의 식염수 수침 전처치가 기관 삽관과 연관된 기도의 합병증에 미치는 영향을 알아보고자 한다. |
| --- | --- |
| 연구 기간 | IRB승인일 ~ 14개월까지 |
| 연구 대상(시험약 등) | Normal saline |
| 연구 대상자 수 | 총 300명 |
| 취약한 연구대상자 | 없음 |
| 연구 방법 | 환자를 무작위로 두군으로 나누어 한군은 기관내 삽관전 멸균 식염수 통에 기관튜브를 미리 담궈두고, 다른 한군은 이런 전처치 없이 기관튜브를 준비한다.  전신 마취 유도 후 직접후두경 하에 준비된 기관튜브로 기관 내 삽관을 시행한다. 두군 사이에 수술 후 후두동통, 애성, 구강 손상등 삽관과 연관된 기도 합병증에 차이가 있는지를 비교한다. |
| 유효성 평가 | Primary end point: 두군 사이의 수술 후 24시간 사이의 후두동통에 차이가 있는지 비교한다. |
| 안전성 평가 | 본 시험에 참여한 모든 환자를 대상으로 안전성 평가를 실시한다.  연구 시행 중에 이상반응이 발생했을 경우, 연구에 사용된 시험 방법과의 인과 관계 유무와 모든 이상 반응을 기록하고 추후 중증도, 중대성, 기간, 그리고 시험 방법과의 인과관계를 평가한다. 이상반응에 대한 처치 및 결과 역시 기록한다. |
| 기대효과 및  예상결과 | 식염수 수침 전처치 없이 기관 삽관을 시행하는 경우 전처치후 시행하는 경우에 비해 기도 합병증이 증가하지 않을 것이다. |

**CONSORT flow diagram**

| 연구 참여 조건을 만족하는 환자 수 (n= ) |
| --- |

Exclusion (n = )

| 무작위로 2군에 배당 (n= 300 ) |
| --- |

| **식염수 전처치 후 기관내 삽관 시행함(n= 150 )**  배정된 방법으로 삽관 (n= )  배정된 방법으로 삽관하지 않음 (n= ): | **전처치 없이 기관내 삽관 시행함(n= 150 )**  배정된 방법으로 삽관 (n= )  배정된 방법으로 삽관하지 않음 (n= ): |
| --- | --- |

| 결과 분석에 포함 (n= )  결과 분석에서 제외 (n= ): | 결과 분석에 포함 (n= )  결과 분석에서 제외 (n= ): |
| --- | --- |

**1. 연구 제목**

기관 튜브의 관습적 식염수 수침 전처치가 기관 삽관 후 합병증에 미치는 영향

(영문) Effect of normal saline immersing of endotracheal tube on post-intubation complications: A prospective randomized study

**2. 연구의 실시기관명 및 주소**

서울대학교 의과대학 마취통증의학과 교실, 서울특별시 종로구 연건동 28

**3. 연구의 책임자 및 담당자**

**3.1. 연구책임자**

서정화

기금부교수

서울특별시 종로구 연건동 28, 서울대학교 마취통증의학과

전화번호: 02-2072-0641

**3.2. 연구담당자**

김유진

진료교수

서울특별시 종로구 연건동 28, 서울대학교 마취통증의학과

전화번호: 02-2072-0779

**4.연구 의뢰기관**

**4.1 연구 의뢰기관 명칭 및 주소** : 해당 없음

**4.2 모니터요원 성명 및 직명**

김태경

진료교수

서울특별시 종로구 연건동 28, 서울대학교 마취통증의학과

전화번호 : 02-2072-2469

**5. 연구비 지원기간 명칭 및 주소 :** 해당 없음

**6. 예상연구기간 :** IRB 승인일 이후부터 14개월까지

**7. 연구 대상 질환** : 전신 마취 하에서 기관삽관하에 수술을 받기로 예정된 환자

**8. 연구 배경 및 목적**

**8.1. 연구 배경**

진공상태에서 밀봉된 기관튜브를 멸균생리식염수에 미리 물에 담궈두었다가 기관삽관을 하는 ‘식염수 수침 전처치’ 방법은 기관내 삽관전에 관습적으로 널리 시행되고 있다. 이 방법은 튜브 커프에 묻은 물이 윤활작용을 하여 기관 삽관으로 인한 기도 관련 합병증을 줄일 수 있을 것으로 예상하기 때문인데, 이에 대한 명확한 과학적 근거는 없으며 실제 임상에서는 수침법과 개봉 직후 사용하는 방법 모두 사용되고 있는 실정이다. 그러나, 과거 Stock등의 연구에 따르면[^1^](#_ENREF_1) dry tube나 normal saline으로 수침한 경우 술후 후두동통이나 애성, 성대손상등의 기도 관련 합병증에 큰 차이가 없는 것으로 보고된바 있으며 멸균되었다고는 하나 개봉후 외부에 노출되어 균의 번식으로 인한 호흡기계 감염의 위험성이 있으며 준비과정이 번거로울수 있는 등 불필요하게 식염수 수침 전처치를 하고 있는지에 대한 의문이 제기되었다.

**8.2 연구 가설 및 목적**

저자들은 이에 식염수 수침 전처치 방법을 시행할 때와 시행하지 않을 때 합병증의 발생률을 비교하고자 한다. 본 연구의 가설은 “식염수 수침 전처치 방법을 사용하지 않는 경우 사용하는 경우에 비해 수술 후 24시간 동안의 후두동통 발생률이 증가하지 않을 것이다”이다.

**9. 임상시험용 의약품 및 의료기기 코드명(또는 주성분의 일반명), 원료약품의 분량,제형 등(대조약 포함)**

크린조® 1000mL (sodium chloride 9g, 제이더블유중외제약㈜, Korea)

**10. 연구대상자의 선정 기준, 제외기준, 목표한 대상자 수 및 산출 근거**

**10.1 선정기준**

전신 마취 하에서 기관삽관이 예정된 20-80세의 성인 환자

**10.2 제외기준**

시험에 동의하지 않거나 시험내용을 이해하지 못하는 환자

위식도역류의 과거력이 있는 환자

아래와 같은 선천적 혹은 후천적인 상기도의 병변이 있는 환자

(ex. 종양, 폴립, 외상, 농양, 염증, 감염, 이물질 등)

기도 관련 수술을 받은 병력이 있는 환자

기도 흡인의 가능성이 증가되어있거나 혈액응고이상이 있는 환자

이전 수술에서 difficult intubation이었거나 일 것으로 예상되는 환자 (Mallampati class 3이상, Thyromental distance 6cm 미만, cervical disease등)

치아 상태 불량으로 lightwand나 fiberoptic bronchoscopy 사용이 필요한 환자

Neck surgery를 받는 환자

수술전 후두동통이나 상기도 감염의 증상이 있는 환자

위식도관을 가지고 있거나 삽입이 필요한 수술을 받는 환자

비강으로 기관내 삽관을 하는 환자

스테로이드나 NSAIDs 정주치료 혹은 투약을 하고 있는 환자

**10.3 목표한 대상자 수 및 산출 근거**

기존의 Borazan등의 연구[^2^](#_ENREF_2)에 의하면 통상적인 방법으로 기관내 삽관을 시행한 환자에서 술후 기관발관후 후두동통 발생률을 57%로 보고하였다. 본 연구에서 식염수 수침 전처치 방법을 시행하지 않은 경우 (dry intubation), 시행한 경우에 비해 후두동통발생률이 증가하지 않을것으로 가정하고, 알파값 0.05, power 0.8, 비열등성인정한계를 15%로 설정하였다. 이에 각 군당 최소 135명씩 필요할것으로 계산되었고 약 10%의 탈락율을 고려하여 각 군당 150명씩, 총 300명의 환자가 필요할 것으로 생각된다.

**10.4 연구 대상자 모집 계획**

전신마취하에서 기관내 삽관 후 수술을 받는 20-80세 사이의 성인환자들 중 10.1 과 10.2의 선정/제외기준에 부합되고 연구대상자가 동의할 경우 대상자로 선정한다. 수술 입원전 외래에서 혹은 입원 전일 환자에게 별첨된 설명문과 동의서에 따라 연구 담당자 혹은 공동 연구자가 환자에게 설명한 후 서면 동의를 받는다.

**11. 연구 방법**

**11.1 구체적인 연구방법**

기관내 삽관 방법을 제외한 모든 임상 처치는 두 군 모두 동일하게 시행한다. 환자들은 전처치 없이 수술실에 입실하여 비침습적 표준 환자 감시장치(심전도, 혈압, 산소포화도) 를 부착한다. 전처치 군 (saline group)에서 삽관 튜브는 환자의 입실 직전 개봉한 멸균 생리 식염수 (크린조® 1L)에 튜브를 미리 담궈두고, 전처치를 시행하지 않는군에서는 (dry group) 기도 삽관 직전 튜브를 밀봉상태에서 튜브를 개봉하도록 한다 (여성에서는 내경 7.0, 남성에서는 8.0의 삽관튜브를 사용한다).

Propofol 1.5-2 mg/kg, FTN 1 mcg/kg 로 마취 유도 후 rocuronium 0.6-0.8mg/kg를 투여받는다. 2분간 facial mask로 용수환기 후, 10년 이상의 기관 삽관 경험을 가진 마취과 전문의가 환자의 머리를 sniffing position으로 유지한 후 Macintosh laryngoscope (#3: female, #4: male)을 구강내로 진입시키고 후두 및 성대를 확인하여 Cormack grade(4-point-scale)를 평가한다.[^3^](#_ENREF_3)

I: no difficulty

II: only posterior extremity of the glottis visible

III: only the epiglottis visible

IV: no recognizable structures visible without laryngeal manipulation

미리 배정된 군에 따라 준비된 삽관튜브를 기도내로 진입시키는데 tube가 glottis를 통과할 때 resistance를 다음과 같이 3-point-scale로 평가한다 (non/mild/moderate). 이후, 호기말 이산화탄소 분압측정으로 기관내 삽관을 확인한 후 양쪽 폐음이 잘 청진되는 위치에 튜브를 고정시킨다. 보조자는 intubation에 소요되는 시간(laryngoscope이 입에 들어가서 나올 때까지 걸리는 시간)과 intubation duration (intubation 직후부터 extubation때까지 걸리는 시간)을 측정한다. 동일한 시술자가 만약 3번의 시도에도 기관내 삽관이 되지 않으면 다른 마취과 의사의 도움을 요청하거나 굴곡하 기관지내시경술, 비디오 후두경술 등을 시행하여 기관내 삽관을 하도록 하며, 증례기록지에 이를 기록한다. 수술중 intra-cuff pressure는 25cmH_2_O로 유지 하며 마취유지는 desflurane 1.0-1.5MAC을 유지하도록 한다.

수술 종료 후 환자의 근이완 정도를 TOF를 사용하여 평가 한 후 reverse를 투여한다. 환자의 자발호흡이 돌아오고 obey command가 가능할 때 extubation을 시행하고 수술종료시점부터 발관까지 걸린 시간과 환자의 구강내나 삽관 튜브의 혈흔여부를 확인한다. 회복실 퇴실 직전과 종료후 2시간, 4시간, 24시간째에 연구대상자의 배정군을 눈가림한 평가자가 병실에서 환자를 진찰하여 구강 인두손상정도와 후두동통 여부를 체크하고 그 점수를 기록한다. 후두동통은 기존 연구의 기준에 따라 4-point-scale로 각각 평가한다.[^2^](#_ENREF_2)

|  | **Sore throat** |
| --- | --- |
| none | No sore throat |
| mild | Mild sore throat (complained of sore throat only upon inquiry) |
| moderate | Moderate sore throat (complained of sore throat on his/her own) |
| severe | severe sore throat (change of voice or hoarseness, associated with throat pain) |

구강 인두 손상은 술후 2시간째와 24시간째에 이학적 관찰로 확인한 후 손상이 있다면, 기존 논문들에서처럼[^4^](#_ENREF_4)^,^ [^5^](#_ENREF_5) 손상부위를 posterior pharyngeal wall, uvula, vallecular, tosillar fossa and pillars로 나누고 hyperemia, edema, hematoma, 기타 등으로 손상정도를 묘사하여 기록한다.

수술 종료 일주일 경과 후 환자에게 문진하여 상기도감염이나 폐렴, 편도선염등 감염 관련 합병증이 발생하였는지 여부를 확인하고 증상 및 정도, 기간 등을 기록한다.

**11.2 비교군 설정 및 무작위 배정 방법**

시험군 (dry group): 식염수 수침 전처치 없이 기관내 삽관을 시행하는 군 150명

대조군 (saline group): 식염수 수침 전처치 후 기관내 삽관을 시행하는 군 150명

연구대상자 등록 전 환자의 진료에 관여하지 않은 의사가 대조군(A)과 시험군(B)을 2명씩포함하는크기가 4인블록과 각각 3명씩 포함하는 크기가 6인 블록이 무작위로 섞인 블록 무작위배정표를 난수표에 따라 미리 작성하고 이 배정표에 따른 순서대로 연구대상자를 대조군이나 시험군에 배정하여 시험을 진행한다. 무작위 배정 비율은 시험군과 대조군에 1:1로 배정되도록 한다. 단, 무작위배정표는 연구대상자가 시험에 등록되기 전에 연구에 참여하는 연구대상자를 볼 수 없는 연구와 독립된 제 3자에 의해 만들도록 하며 운영, 관리되어 Allocation concealment를 유지하도록 한다. 연구에 포함되는 총 300명의 환자의 배정표가 작성되면 제 3자가 무작위배정표를 보관하고 있다가 연구자가 전일 스크리닝하여 대상으로 산정한 환자를 배정표 순서에 따라 매칭하여 연구자에게 알려주도록한다.

**11.3**

**시험약 투여∙사용량, 투여∙사용 방법, 병용 요법, 대조약 사용시 그 선택사유** : 해당없음

**11.4 관찰 항목, 임상검사항목 및 관찰검사방법**

- 환자의 인구학적 자료: 성별, 나이, 체중, 신장

- 환자의 modified Mallampati class, Cormack-Lehane grade,

- 마취약제 투여후 용수환기중 마스크 환기의 용이도 (easy/moderate/difficult)

- 기관내 삽관 시도중 삽관시간, 삽관유지시간, 삽관 저항도

- 삽관 전후의 혈역학적 변화: 혈압, 심박수, 산소포화도

- 수술 후 발관까지 걸린 시간

- 발관 후 환자의 구강내나 삽관튜브의 혈흔, 삽관튜브 커프의 손상여부

- 회복실, 2시간, 4시간, 24시간째 환자의 애성, 후두동통, 2시간, 24시간째 환자의 구강인두 손상 정도

- 수술후 사용된 진통제 종류 및 양 (PCA포함)

- 수술후 1주일째까지 상기도감염, 폐렴, 편도선염 등의 기도감염관련 합병증 유무

- 기타 event

**11.5 효과 평가기준, 평가 방법**

Primary end point: 두군 사이의 술후 후두동통 여부를 비교한다.

**11.6 기존 치료 및 연구와의 차별점**

기존의 연구에 따르면 normal saline으로 수침 전처치한 군과 dry tube를 사용한 군에서 술후 후두동통에 있어서 차이가 없었음을 보고한 바 있다.[^1^](#_ENREF_1) 그러나 기존의 연구는 환자의 기대효과에 따른 샘플수 산정을 하지 않고 단순 비교하였고 primary outcome도 dry group과 saline group을 비교한 것이 아니라 lidocaine jelly 처치 후의 effect를 비교한 것이었다.

본 연구는 양군간 차이가 없음을 증명하기에 적합한 비열등성 검정 모델을 채택하여 샘플수를 산정하였고, 수술 후 호흡기계 감염 합병증 비교를 추가하여 기존의 연구에 비해 차별성이 있다 여겨진다.

**11.7 연구대상자의 이익과 위험**

일반적으로 기관내 삽관시에는 항시 삽관지연으로 인한 저산소증, 구강 점막의 손상 및 미세 출혈, 삽관 튜브 커프의 손상, 성대 손상, 치아 손상, 후두 동통, 애성, 호흡기계 감염등의 합병증의 위험성이 있다. 본 연구에서 사용하는 식염수 수침 전처치나 dry tube 방법 모두 임상에서 널리 사용되는 방법이며, 기존의 소규모 스터디에서 두 방법간 수술 후 합병증 차이점이 없는 것으로 나타났으며 전신 마취하에 기관내 삽관이 필요한 수술을 받는 환자에서 연구를 진행하므로, 본 연구로 인해 추가적인 위험이 생길 여지는 적다고 생각된다. 또한 모든 과정은 숙련된 마취과 전문의가 곁에서 감시하며 합병증이 발생하더라도 적절한 조치를 취하게 되므로 환자에게 추가 위험의 가능성은 매우 적을 것으로 생각된다.

**11.8 중지, 탈락 기준**

연구대상자의 동의 철회

표준적인 후두경하 기관내 삽관이 어려워 다른 기구로 기관내 삽관을 한 경우

수술 후 바로 extubation을 하지 못하는 경우

예기치 않게 수술 중 L-tube를 넣어야 하는 경우

**11.9 부작용을 포함한 안전성의 평가기준, 평가 방법 및 보고 방법**

본 시험에 참여한 모든 환자를 대상으로 안전성 평가를 실시한다.

연구 시행 중에 이상반응이 발생했을 경우, 연구에 사용된 시험 방법과의 인과 관계 유무와 모든 이상 반응을 기록하고 추후 중증도, 중대성, 기간, 그리고 시험 방법과의 인과관계를 평가한다. 이상반응에 대한 처치 및 결과 역시 기록한다. 이상 반응은 시험 기간 중의 계획된 검진과 검사의 소견 이외에도 비정상적인 검진이나 필요에 따른 추가적인 검사와 검진에 의해 평가하고 즉각적인 조치를 시행한다. 임상병리검사 자료에 대해서는 변수의 특성에 따라 치료 전, 후의 군내 비교 등 적절한 통계적 방법을 이용하여 분석하고, 이상반응의 빈도, 발현율, 각각의 목록, 심각한 정도 및 시험 방법과의 인과관계 등을 제시하며, 필요한 경우 그래프 형태로 보고한다.

1)중증도

이상반응은 아래의 정의에 따라 경증, 중등증, 중증으로 구분된다.

경증은 일반적이고 일시적이고 일상적인 활동을 방해하지 않는다.

중등증은 약간의 불편함을 초래하거나 일상적인 활동을 방해한다.

중증은 일상적인 활동을 수행할 수 없다.

2)인과 관계

인과 관계는 ‘관련 없을 것으로 생각됨’, ‘관련 있을 가능성 있음’, ’가능성 많음’, ’명백히 관련 있음’, 또는 ‘관련성을 확인하기 어려움’으로 구분된다.

3)이상 반응의 분류

기도 내 삽관과 관련된 이상 반응

저산소증, 구강 점막의 손상 및 미세출혈, 삽관 튜브 커프의 손상, 성대손상, 치아

손상, 애성, 후두 동통 등의 합병증이 발생 할 수 있으나 이는 기존의 통상적인 후두경하

기관내 삽관시 발생할 수 있는 합병증이며, 본 연구로 인하여 추가적인 위험이 생길

여지는 적다.

**11.10 자료 및 안전성 모니터링 계획(DSMP)**

모니터링 위원 : 김태경

모니터링 위원의 소속 및 지위 : 마취통증의학과 진료교수

연락처 : 02-2072-2469

자료의 안전성 정보 수집 및 검토 주기 : 6개월마다, 연구 대상자의 모집 완료 후

연구의 지속, 변경, 중단 결정을 위한 주요 유효성 평가 변수 및 검토 절차 :

총 300명의 연구대상자 중 절반인 150명이 모였을 때 일차 유효성 평가 변수인 수술 후 후두동통에 있어서 양군간의 차이가 40% 이상으로 저명하게 나타날 경우 연구를 지속함이 윤리적으로 타당하지 않다고 보고 IRB에 보고 후 연구를 변경 혹은 중단하도록 한다.

규제기관에 대한 보고 절차

: 환자에게 주술기에 예기치 않은 중대한 이상반응 (사망, 중환자실 입실 등)혹은 계획서 미준수등이 발생할 경우 추가 모집을 일시 중단하고 2주 안에 IRB에 보고하여 논의를 거쳐 연구의 지속, 변경 여부를 검토하도록 한다. 이후 연구와 이상반응이 무관한 것으로 밝혀질 경우 연구를 진행하도록 한다.

본 연구의 모니터링은 정기적으로 시험기관을 방문하여 연구대상자 모집, 연구대상자 등록, 데이터 저장 및 분석에 대한 평가, 증례기록서에 기입된 정보의 정확성, 임상시험계획서와 GCP의 준수 여부를 확인할 것이다. 본 연구는 병원윤리위원회의 윤리규정을 준수할 것이다.

**11.11 자료 분석 및 통계 분석 방법**

통계분석에 포함 시킬 분석군은 full analysis set로 중대한 결측치 (primary outcome인 후두동통 혹은 애성 미기입)가 있거나 선정기준 및 제외기준에 맞지 않는 환자가 참여하였음을 나중에 발견하게 되는 경우 통계분석에서 제거하며, 임상시험 결과 보고서에 제외시 연구대상자들이 왜 제외되었는지 그 이유를 쓰고 요약하도록 한다. 연구대상자 분석군은 대조군과 시험군 2군으로 나누어 양 군을 비교하고자 한다.

1. Non-inferiority test를 통해 양군간 수술 후 24시간 동안의 후두동통 발생률을 비교분석한다.
2. 환자의 인구학적 자료나 삽관에 걸리는 시간, 삽관을 유지한 시간, 술 후 발관까지 걸린 시간, 삽관 전후의 혈역학적 변화, 술 후 후두동통, 애성등 연속적 변수에 대해 정규성 검정을 통해 정규분포를 하는 것으로 확인되면 student t-test를, 정규분포를 하지 않으면 Mann Whitney U-test를 이용하여 군간 비교를 시행한다.
3. 환자의 modified M class, Comarck grade, 마스크 환기 용이도, 삽관 저항도, 환자의 구강내나 삽관튜브의 혈흔, 삽관튜브 커프의 손상여부, 구강인두 손상 여부, 수술 후 1주일째 확인한 기도감염관련 합병증 유무에 대해 chi-square test 혹은 Fisher’s exact test를 이용하여 분석한다.

통계 프로그램은 SPSS 21.0 (SPSS, Chicago, IL, USA)을 사용하며 모든 평균 값들은 평균±표준편차로 표시한다.

11.12 연구수행일정표

2015년 IRB 통과 직후 14개월

2015년 IRB 통과 직후 1개월 : 시험준비

2015년 IRB 통과 1개월부터 13개월 : 임상시험

2015년 IRB 통과 13개월 후부터 1개월 : 자료 정리 및 논문 작성

**12. 연구 대상자의 안전보호를 위한 대책**

**1) 연구의 윤리성 확보를 위한 기본 방안**

2008 헬싱키 선언 (2013년 개정)에 입각하여, 연구대상자 또는 보호자에게 연구의 목적과 연구 참여 중 일어날 수 있는 정신적, 신체적 위해를 충분히 설명한 후 연구대상자(또는 보호자) 로부터 서면동의서를 받을 예정이다. 연구대상자, 연구대상자의 담당의사, 시험 참여자 이외에는 연구대상자의 시험 참여 여부나 치료 경과에 대해 알지 못하게 하며, 연구대상자의 신원을 파악할 수 있는 기록은 비밀로 보장될 것이다. 연구를 위해 수집되는 정보는 잠금장치가 있는 연구실에 비밀번호가 걸린 파일로 보관하고 연구파일에 접근할 수 있는 사람은 권한을 가진 일부 연구원으로 제한할 것이다. 수집되는 자료의 불필요한 개인식별자는 제거하고, 특히, 증례기록서에는 환자의 이름, 주민등록번호, 차트 번호 등을 기재하지 않도록 하며, 신상정보와 연결된 식별자 코드는 별도로 관리할 것이다. 환자와 관련된 사진을 제출할 때는 환자의 신원을 알 수 없도록 할 것이며 조금이라도 신원이 노출될 가능성이 있는 경우에는 이에 대한 서면 동의를 받았음을 명시할 것이다. 연구대상자의 검진 기록은 비밀이 유지되고 다른 곳으로 이동되지 않을 것이며, 본 연구의 진행 여부를 감독 받기 위해 감독 기관으로 보내어질 수 있다. 그리고 이 자료는 법이 정하는 기간 동안 보관될 것이며 추후 모든 자료는 폐기될 것이다. 본연구는 ICH-GCP를 준수할 것이며 적법한 절차에 따라 IRB 승인 후 연구를 수행할 것이다.

**2) 연구대상자의 동의 과정**

GCP 교육을 이수한 연구 담당자가 연구대상자 또는 보호자에게 연구의 목적과 연구 참여 중 일어날 수 있는 정신적, 신체적 위해를 충분히 설명한 후 연구대상자(또는 보호자) 로부터 서면동의서를 받을 예정이다. 연구대상자, 연구대상자의 담당의사, 시험 참여자 이외에는 연구대상자의 시험 참여 여부나 치료 경과에 대해 알지 못하게 하며, 연구대상자의 신원을 파악할 수 있는 기록은 비밀로 보장될 것임을 자세히 설명하도록 한다.

**3) 연구대상자의 보상 방안**

시험에 참여 여부에 관계없이 추가적인 금전적, 물질적 보상은 없으며 마찬가지로 환자에게도 추가적인 비용증가는 없을 것이다.

**4) 연구 대상자의 개인정보보호 방안**

연구대상자의 의무기록을 포함한 사적정보의 보호를 위해 다음의 내용을 실천한다.

① 데이터는 미리 준비한 case report form에 기록하며, 즉시 잠금장치가 있는 연구실에 비밀번호가 걸린 파일로 보관한다.

② 연구파일에 접근할 수 있는 사람은 권한을 가진 일부 연구원으로 제한한다. (연구원의 소속: 마취통증의학과, 성명: 서정화, 김유진)

③ 수집되는 자료의 불필요한 개인식별자는 제거한다. 특히, 증례기록서에는 환자의 이름, 주민등록번호, Chart No. 등을 기재하지 않도록 하며, 신상정보와 연결된 식별자 코드는 별도로 관리한다.

④ 생명윤리법 시행규칙 제 15조에 따라 연구 관련 기록을 연구가 종료된 시점부터 3년간 보관하여야 하며, 보관기간이 지난 문서는 개인정보보호법 시행령 제16조에 따라 파기한다. 다만 후속 연구, 기록, 축적 등을 위해 3년 이상 보관시 해당 사항을 IRB에 추후 보고하도록 한다.

**13. 인체유래물의 보관 및 폐기 방법** : 해당없음

**14. 참고문헌**

1 Stock MC, Downs JB. Lubrication of tracheal tubes to prevent sore throat from intubation. *Anesthesiology* 1982; **57**: 418-20

2 Borazan H, Kececioglu A, Okesli S, Otelcioglu S. Oral magnesium lozenge reduces postoperative sore throat: a randomized, prospective, placebo-controlled study. *Anesthesiology* 2012; **117**: 512-8

3 Cormack RS, Lehane J. Difficult tracheal intubation in obstetrics. *Anaesthesia* 1984; **39**: 1105-11

4 Chang JE, Min SW, Kim CS, Lee JM, No H, Hwang JY. Effect of Jaw Thrust on Transesophageal Echocardiography Probe Insertion and Concomitant Oropharyngeal Injury. *J Cardiothorac Vasc Anesth* 2015

5 Seo JH, Kwon TK, Jeon Y, Hong DM, Kim HJ, Bahk JH. Comparison of techniques for double-lumen endobronchial intubation: 90 degrees or 180 degrees rotation during advancement through the glottis. *Br J Anaesth* 2013; **111**: 812-7
