# Supplementary material for: Tracheal tubes lubricated with water to reduce sore throat after intubation: A randomized non-inferiority trial
Source: PLoS One. 2018 Oct 4;13(10):e0204846. doi: 10.1371/journal.pone.0204846 (PMC6171884; doi:10.1371/journal.pone.0204846)
Supplement: S2 Protocol — (DOCX) [file pone.0204846.s003.docx]

**Clinical Study Protocol**

**(Version 2.0)**

**1. Study Title**

Effect of normal saline immersing of endotracheal tube on post-intubation complications: A prospective randomized study

**2. Address of Research Center**

Seoul National University Hospital, Seoul National University College of Medicine, 101 Daehak-ro,

Jongno-gu, Seoul 110-744, Republic of Korea

**3. Principal investigators and Co-investigators**

3.1 Principal Investigators

Jeong-Hwa Seo

Associate professor

Department of Anesthesiology and Pain Medicine, College of Medicine, Seoul National University

3.2 Co-investigator

Eugene Kim

Clinical professor

Department of Anesthesiology and Pain Medicine, Seoul National University Hospital

**4. Responsibility of trial**

Investigator-initiated trial

**5. Address of sponsorship**

None

**6. Study period**

14 months after the approval of IRB

**7. Subjects of study**

who are scheduled for elective surgery under general anesthesia with orotracheal intubation

**8. Necessity of Study and Overview**

8.1 Background

In clinical field, water is commonly used for lubrication of tracheal tubes before tracheal intubation. Because its known lubricating properties, many clinicians expect the water can decrease airway injuries caused by intubation. However, this conventional pretreatment seems to have been performed without any validation for its advantage.

Besides, the wet condition of the tube may trigger the proliferation of respiratory pathogens, thereby increasing the risk of respiratory infection. Moreover, any external treatment applied to the tracheal tube before intubation can lead to contamination of the tube as opposed to keeping the tube inside the sterile packing.

8.2 Hypothesis and objectives

Therefore, we would conduct this study to investigate whether no pretreatment of tracheal tubes does not increase post-intubation airway-related complications as compared with water lubrication of tubes in patients undergoing general anesthesia. Our hypothesis is that a tube lubricated with water does not reduce sore throat after tracheal intubation compared to a tube without lubrication

**9. Study materials**

Klenzo 1000 mL (sodium chloride 9g, JW medical™, Korea)

**10. Inclusion and exclusion criteria**

10.1 Inclusion criteria

Participants with American Society of Anesthesiologists physical status 1–3 and aged 20–80 years, who are scheduled for elective surgery under general anesthesia with orotracheal intubation

10.2 Exclusion criteria

- Symptoms of sore throat, hoarseness, and respiratory infections as assessed by a study

investigator at baseline

- Gastroesophageal reflux diseases defined by history taking from patients

- Congenital or acquired abnormalities of the upper airway

- Previous airway surgeries

- Previous history of aspiration

- Coagulation disorders

- History of difficult intubation or conditions with an expected difficult airway including Mallampati classification ≥3 or a thyromental distance <6.5 cm

- Use of airway instruments other than a direct laryngoscope such as a fiberoptic bronchoscope,

video laryngoscope, or lighted stylet

- Anticipated nasotracheal intubation or insertion of a nasogastric tube

10.3 Sample size calculation

The primary outcome of this trial is the incidence of postoperative sore throat within 24 h after surgery. Assuming the incidence of 57% in a previous study, 135 patients are required in each group to obtain 80% statistical power, 5% risk of type-I error, and 15% noninferiority margin. The margin was determined based on our clinical judgment that the incidence of postoperative sore throat is relatively high (50–60%), so the difference within 15% in the incidence would be considered clinically noninferior. Considering 10% dropout, the estimated sample size is 300 in both groups. The sample size is calculated using a PASS software (version 11.0, NCSS, Kaysville, UT, USA).

10.4 How to recruit

Potential participants who meet the inclusion/exclusion criteria (See 10.1 and 10.2) are recruited at outpatient clinics or on the preoperative visit before surgery, and written informed consents are obtained from all of the participants.

**11. Study protocols**

11.1 Interventions

After overnight fasting, participants enter the operating room without any premedication and standard monitoring (electrocardiography, noninvasive arterial blood pressure, and pulse oximetry) are applied. According to random allocation, a disposable tracheal tube (Unomedical, Kedah, Malaysia), which is made of polyvinyl chloride and has a cuff with high-volume and low-pressure characteristics, is pretreated by an anesthesia nurse unaware of the study protocol. Until

tracheal intubation, the tube is placed in a 1-L bottle of sterile saline for the experimental group or kept inside the sterile packing for the control group. Tubes with 7.0-mm and 7.5-mm internal diameters are used for women and men, respectively

General anesthesia is induced with intravenous administration of propofol 1.5–2.0 mg/kg and fentanyl 1 mcg/kg. Rocuronium 0.6–0.8 mg/kg is administered for the neuromuscular blockade and train of-four counts are checked at the adductor pollicis muscle using acceleromyography (TOF-watch®, Organon Ltd., Dublin, Ireland). At a train-of-four count of 0, the tracheal tube, pretreated according to the randomization, is handed to an investigator who has a 10-year experience in tracheal intubation. The investigator performs tracheal intubation via direct laryngoscopy using either Macintosh 3 or 4 blades and evaluates the Cormack-lehane grade.

I: no difficulty

II: only posterior extremity of the glottis visible

III: only the epiglottis visible

IV: no recognizable structures visible without laryngeal manipulation

The intubation practitioner subjectively evaluates the resistance during advancement of the tube through the glottis using a four-point scale (none, mild, moderate, and severe). Intubation time, defined as the duration between the insertion of the laryngoscopic blade into the mouth and the inflation of the endotracheal tube cuff, is measured. If intubation fails, the tube tip is flexed to make a hockey-stick shape by inserting the stylet inside the tube, and then intubation is reattempted. When intubation fails in 3 attempts even using the stylet, other devices such, as a video laryngoscope, fiberoptic bronchoscope, or lighted stylet, are applied. Intracuff pressure is maintained at 25 cmH_2_O. Anesthesia is maintained with 1.0–1.5 minimum alveolar

concentration of desflurane to obtain a bispectral index (A2000 XP, Aspect Medical Systems, Newton, MA, USA) value of less than 60, and total fresh gas flow is supplied at 2 L/min throughout the operation.

At the end of surgery, reversal agents are administered to antagonize the neuromuscular blockade. After gently suctioning the oral secretions from the oropharynx, extubation is carefully performed at a train-of-four ratio above 90% when the participants are able to achieve spontaneous breathing and obey verbal commands. Extubation time is defined as the interval from cessation of anesthesia until extubation. After extubation, the presence of blood on the tube surface or in the oral cavity is examined. An investigator blinded to the group assignment evaluates patient-reported sore throat at 0, 2, 4, and 24 h after surgery.


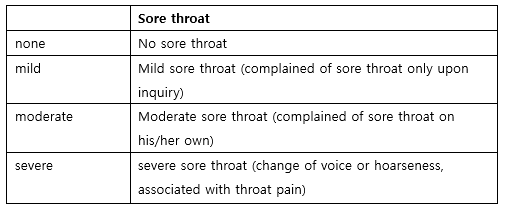


The investigator also examines oropharyngeal injuries via direct inspection using a penlight and tongue depressor with regard to the location (posterior pharyngeal wall, uvula, tonsillar fossa, pillar, or others) and type (hyperemia, edema, hematoma, or others) at 2 and 24 h after surgery. The amount of intraoperative and postoperative analgesic drugs is checked until 24 h after surgery.

At 7 days after surgery, the investigator also asks about the symptoms of respiratory infections, such as cough, sputum, rhinorrhea, sore throat, or fever, and whether the patients have been diagnosed with a common cold, tonsillitis, pneumonia, or any other respiratory infectious diseases and prescribed related medications postoperatively. If the patients have been discharged from hospital, they are contacted by telephone and asked about respiratory infections.

11.2 Randomization and Blinding

Experimental group (dry group): tracheal intubation without pretreatment (n = 150)

Control group (saline group): tracheal intubation with water lubricated tube (n = 150)

After the recruitment, participants are randomly assigned to one of the two groups in a 1:1 ratio depending on whether or not the tracheal tube is lubricated with water before intubation.

A random sequence with 4 or 6 sizes of random blocks (i.e., 4-4-4-6-4-4-6-6…) is generated

with an online tool by an assistant not involved in the trial and kept within sealed opaque envelopes. When a patient is enrolled in the trial, an anesthesia nurse opens an envelope and prepares a tracheal tube with the allocated treatment.

11.3 Administration and dose of drug: N/A

11.4 Outcome measurements

- Demographic data: sex, age, body weight, height, body mass index

- airway related parameters: modified Mallampati class, Cormack-Lehane grade, thyromental distance

- ease of mask ventilation (easy/moderate/difficult)

- intubation time, duration, resistance of tube advancement

- hemodynamic parameter change before and after the intubation : mean blood pressure, heart rate, saturation

- Extubation time

- oral bleeding, blood tinged tracheal tube cuff, mechanical injury of tracheal tube

- Sore throat and hoarseness at 0, 2, 4 and 24 after the surgery

- Oropharyngeal injuries at 2 and 24 h after the surgery

- Amount of postoperative analgesics

- Symptoms of respiratory infections of diagnosis of any other respiratory infectious diseases up to 7 days after the surgery

11.5 Primary outcome measurements

- Incidence of sore throat during postoperative 24 h

11.6 Expected Adverse Events and Precautions for Use

In general, there is a risk of complications such as hypoxia due to delayed intubation, mucosal injury and microhemorrhage, tracheal tube cuff injury, vocal cord injury, dental damage, laryngeal pain, hoarseness, and respiratory infections. Both the water-lubricated and the dry tube method were widely used in clinical practice, and there was no difference in postoperative complications between the two methods in a previous small-case study. Therefore, it is unlikely that additional risk will arise from this study. In addition, all procedures are monitored by a trained anesthesiologist, and even if complications occur, appropriate treatment will be taken so that the possibility of additional risk to the patient is expected to be minimal.

11.7 Criteria for Discontinuation and Drop-out

- Withdrawal of consent

- Intubation with other alternative airway devices rather than standard laryngoscopy

- In a case of that cannot extubate after the surgery

- In a case of that should insert the nasogastric tube unexpectedly

11.8 Plan for report of harmful cases

Reports of serious adverse events (airway-related problems such as hypoxic brain damage, hypoxic organ damage et etc.) and unexpected problems and research-related adverse events are reported to the IRB within 7 days, and within 24 hours for serious and unexpected adverse events.

11.9 Data Safety Monitoring Plan (DSMP)

Principal investigator will make comparisons between the source documents and the study protocol at every 6 months to assure the completeness of data and will review the safety data of subjects. The subject identification code on the data will be encoded, and will be filed in a safe installed with a locking device while the electronic document will be saved in a computer which is restricted for access.

**12. Statistical analysis**

Both intention-to-treat and per-protocol analyses will be performed and missing data will be imputed with the last observation carried forward value. Continuous variables

will be presented as mean and standard deviation or median and interquartile range according to the Kolmogorov-Smirnov test, and categorical variables as number of patients and proportion.

The incidence of postoperative sore throat (primary outcome)

will be compared with a noninferiority analysis.The noninferiority of the nonpretreated tube over the water-lubricated tube will be accepted if the upper bound of a 95% confidence interval is below the predetermined noninferiority margin of 15%. For secondary outcomes, continuous variables will be compared with an independent t test or the Mann-Whitney U test and categorical variables with Pearson’s chi-squared test or Fisher’s exact test.

All tests are two-sided and P < 0.05 is considered statistically significant. A statistician not involved in data collection will conduct all statistical analyses using SPSS software (version 21.0, SPSS Inc., IBM, Chicago, IL, USA).

**13. References**

1 Stock MC, Downs JB. Lubrication of tracheal tubes to prevent sore throat from

intubation. *Anesthesiology* 1982; **57:** 418-20

2 Borazan H, Kececioglu A, Okesli S, Otelcioglu S. Oral magnesium lozenge reduces

postoperative sore throat: a randomized, prospective, placebo-controlled study. *Anesthesiology* 2012; **117:** 512-8

3 Cormack RS, Lehane J. Difficult tracheal intubation in obstetrics. *Anaesthesia* 1984; **39:** 1105-11

4 Chang JE, Min SW, Kim CS, Lee JM, No H, Hwang JY. Effect of Jaw Thrust on Transesophageal Echocardiography Probe Insertion and Concomitant Oropharyngeal Injury. *J Cardiothorac Vasc Anesth* 2015

5 Seo JH, Kwon TK, Jeon Y, Hong DM, Kim HJ, Bahk JH. Comparison of techniques

for double-lumen endobronchial intubation: 90 degrees or 180 degrees rotation during

advancement through the glottis. *Br J Anaesth* 2013; **111:** 812-7
